# Photoperiod Affects Harderian Gland Morphology and Secretion in Female *Cricetulus barabensis*: Autophagy, Apoptosis, and Mitochondria

**DOI:** 10.3389/fphys.2020.00408

**Published:** 2020-05-06

**Authors:** Zhe Wang, Jin-Hui Xu, Jun-Jie Mou, Xiao-Tong Kong, Ming Wu, Hui-Liang Xue, Lai-Xiang Xu

**Affiliations:** College of Life Sciences, Qufu Normal University, Qufu, China

**Keywords:** photoperiod, Harderian gland, autophagy, apoptosis, mitochondria

## Abstract

Photoperiod is an important factor of mammalian seasonal rhythm. The Harderian gland (HG) appears to act as a “standby” structure of the retinal-pineal axis, mediating light signals *in vitro* and neuroendocrine regulation *in vivo*; however, the effect of photoperiod on the HG is not clear. Here, we studied morphological differences in the HG of female striped dwarf hamsters (*Cricetulus barabensis*), a small mammal that experiences an annual rhythm, under different photoperiods (i.e., SP, short photoperiod; MP, moderate photoperiod; LP, long photoperiod), and further investigated the molecular mechanisms related to these morphological differences. Results showed that body weight, carcass weight, and HG weight were higher in the SP and LP groups than that in the MP group. Protein expression of hydroxyindole-o-methyltransferase, a key enzyme in melatonin synthesis, was higher in the SP group than in the other two groups. Somatostatin showed highest expression in the LP group. Furthermore, comparison of changes in the HG ultrastructure demonstrated autolysosome formation in the SP group. Protein aggregation and mRNA expression of LC3 and protein expression of LC3II/LC3I were higher in the SP group than in the MP group, indicating elevated autophagy under SP. Chromatin agglutination and mitochondrial damage were observed and bax/bcl2 and cytochrome C expression increased at the protein and mRNA levels in the SP and LP groups, suggesting increased apoptosis. Protein expression of dynamin-related protein 1 and mitochondrial fission factor (Mff) were highest in the SP group, suggesting elevated mitochondrial fission. Protein expression levels of adenosine triphosphate (ATP) synthase and citrate synthase were lower in the LP group than in the SP and MP groups. These results indicated that autophagy and apoptosis imbalance under SP and LP conditions may have led to HG weight loss and up-regulation of mitochondrial apoptosis may have weakened mitochondrial function under LP conditions. Finally, melatonin synthesis appeared to be positively correlated with the time hamsters entered darkness.

## Introduction

Seasonal rhythm is an adaptive behavior of temperate-region animals to seasonal variations and includes changes in development, reproduction, hair growth, and energy metabolism (Nakayama and Yoshimura, 2018). Photoperiod (i.e., length of sunshine) is one of the most important factors affecting seasonal behavior (e.g., reproduction) in animals (Borniger and Nelson, 2017). Seasonal rhythm in mammals regulates growth and reproduction by influencing the hypothalamus-pituitary-thyroid gland axis or hypothalamus-pituitary-sexual gland axis through melatonin (MT) (Payne, 1994; Buzzell, 1996; Tsutsui and Ubuka, 2018). In Siberian hamsters (*Phodopus sungorus*), the concentration of MT in blood under different photoperiods differs significantly after 2 h in the dark, peaking at 3 h under long photoperiod conditions (Raiewski et al., 2012). MT is secreted by photosensitive organs such as the pineal gland and Harderian gland (HG) (Payne, 1994; Buzzell, 1996; Tsutsui and Ubuka, 2018). The HG, also known as the glandulae lacrimales accessoriae, is located in the medial orbit and widely exists in mammals, birds, and reptiles with a symmetrical distribution on the left and right (Sakai, 1989; Buzzell, 1996). When rats into the dark 3 h under moderate photoperiod (MP), the concentration of MT is about 200 ng/g in the pineal gland and 6.8 ng/g in the HG, suggesting that the HG may also be involved in regulation of MT under changes in photoperiod (Pang et al., 1977). However, the specific mechanism involved in HG-induced photoperiod signal regulation is not clear.

The secretion of MT in mammals is regulated by two enzymes, namely, arylalkylamine-*N*-acetyltransferase (AANAT) and hydroxyindole-O-methyltransferase (HIOMT) (Simonneaux et al., 2006; Tan et al., 2016), both of which exhibit a circadian rhythm as well as expression in the mammalian HG (Hartmann and Kluge, 1989; Touitou, 2005), where MT receptors (MTRs) are also distributed (Bubenik et al., 1976; Menendez-Pelaez et al., 1993; Guerrero et al., 1994). Short photoperiod (SP) treatment reduces AANAT protein expression in the HG but has no influence on that of HIOMT in female Syrian hamsters (*Mesocricetus auratus*) (inbred LSH/SsLak) (Menendez-Pelaez et al., 1988b), and HIOMT and AANAT expression in the HG remains the same under both SP and long photoperiod (LP) conditions in non-inbred Syrian hamsters (Buzzell et al., 1990). This suggests that there may be differences in the response of MT synthesis to photoperiodic changes in the HG of different mammals. As a major negative regulator of vertebrate growth and development, somatostatin (SS) participates in multilevel regulation of the growth hormone-insulin axis (Sun and Coy, 2016; Morisset, 2017). Research has found that SS mRNA expression in the HG of rats is lower in spring and summer and higher in autumn and winter (Mato et al., 1996). Studies on MT, SS, and MTR in the HG would not only help to clarify the effects of hormone regulation on the HG but also to elucidate the secretory function of the HG under different photoperiods.

Earlier research reported that the secretory functions of the HG in mice are associated with changes in autophagy levels (Koenig et al., 2015). Autophagy is the phagocytosis of cytoplasmic proteins or organelles and their entrapment and degradation in vesicles (Biazik et al., 2015; Dong et al., 2015). The most direct criterion for the occurrence of autophagy is whether bilayer membrane vesicles enclosing substances or organelles can be observed under electron microscopy (Biazik et al., 2015; Dong et al., 2015). As a key protein for autophagic lysosome formation, microtubule-associated protein 1 light chain (LC3-I) binds to the phosphatidylethanolamine (PE) complex to form LC3 II (Lee and Lee, 2016; Schaaf et al., 2016), a protein marker of intracellular macrophages and autophagic changes (Mizushima and Yoshimori, 2007; Schaaf et al., 2016). First discovered in 2013 (Zhang et al., 2013), P62 is a transporter of degradable substances to autophagic lysosomes and is negatively related to autophagy levels in tissues (Lamark et al., 2017). Quantitative analysis of LC3 and P62 proteins can indicate changes in autophagy levels in the HG under different photoperiods. MT can inhibit autophagy in the HG of female Syrian hamsters (Coto-Montes and Tomas-Zapico, 2006; Kongsuphol et al., 2009; Nopparat et al., 2010). To date, however, no research on the effects of photoperiod on autophagy has been conducted.

The secretory activity of the HG is influenced by various exogenous factors (such as light and temperature) and high-intensity light can lead to increased apoptosis in the HG of rats (Kurisu et al., 1996; Monteforte et al., 2008). Bax, which is one of the most important apoptotic molecules in mammals, is activated under high mitochondrial depolarization for translocation and insertion into the outer membrane of mitochondria via bax/bax-homo-oligomerization (Fu et al., 2016). This is followed rapidly by the formation and opening of a mitochondrial permeability transition pore (mPTP), through which cytochrome C (Cyto C), a mitochondrion-residing apoptogenic factor, is released into the cytosol, eventually leading to nuclear DNA cleavage and cell apoptosis (Zha et al., 1996; Smith et al., 2000). At present, DNA fragmentation detected by TUNEL staining is considered one of the most important indicators of increased apoptosis (Smith et al., 2000). Furthermore, bcl2 inhibits apoptosis via suppression of bax/bax-homo-oligomerization (Korsmeyer et al., 1993; Antonsson et al., 1997). Research has shown that injection of MT can promote apoptosis in the HG of Syrian hamsters (Vega-Naredo et al., 2012). Quantitative analysis of apoptosis in the HG can help clarify the mechanisms related to the effects of photoperiodic changes on the morphology and function of the HG.

Changes in apoptotic and autophagic levels often involve mitochondrial function. Citrate synthase (CS) is a limiting enzyme of the tricarboxylic acid cycle (Wiegand and Remington, 1986; Remington, 1992) and adenosine triphosphate (ATP) synthase is a rate-limiting enzyme of the ATP synthesis pathway (Kramarova et al., 2008). Thus, studies on CS and ATP synthase could help clarify the effects of different photoperiods on mitochondrial function. Changes in mitochondrial function may involve mitochondrial fission. Dynamin-related protein 1 (Drp1) is a GTP-hydrolyzing mechanoenzyme that catalyzes mitochondrial fission in the cell, which drives division via GTP-dependent constriction (Kraus and Ryan, 2017; Tilokani et al., 2018). The Drp1 receptor mitochondrial fission factor (Mff) is a major regulator of mitochondrial fission, with its overexpression resulting in increased fission (Tieu and Nunnari, 2000). In contrast, Drp1 receptor fission 1 (Fis1) appears to recruit inactive forms of Drp1, and its overexpression inhibits mitochondrial fission (Liu and Chan, 2015; Yu et al., 2019). Studies on these three factors could highlight mitochondrial fission ability; however, there is a current lack of research on mitochondrial fission and the function of HG during different photoperiods.

Based on the above, the effects of photoperiod on the HG may be related to autophagy, apoptosis, and mitochondrial function. Current photoperiod studies in hamsters have mainly focused on changes in HG morphology (Menendez-Pelaez et al., 1988a, b; Buzzell et al., 1990). However, the mechanisms involved in the morphological changes of the HG induced by different photoperiods, such as autophagy and apoptosis, as well as the secretion of growth-related hormones in small mammals during different photoperiods, remain poorly studied. The striped dwarf hamster (*Cricetulus barabensis*) is a small seasonal rhythmic mammal widely distributed in northern Chinese farmland with high reproductive capacity. These hamsters show peak reproductive activity in spring (March to April) and autumn (August to September), but none during winter (December to January) (Zhao et al., 2010; Xu and Hu, 2017). Our previous research on the striped dwarf hamster showed significant seasonal changes in the expression of hypothalamic genes (e.g., *kiss1* and *gpr54*) and the regulation of immune function and energy metabolism (Zhao et al., 2014). Thus, research on photoperiodic changes in this species could provide further insights into seasonal rhythm changes in non-hibernating mammals.

Here, we studied the morphological and secretory changes, as well as the related mechanisms, in the HG of striped dwarf hamsters under different photoperiods. We hypothesized that photoperiodic changes would affect the morphology of the HG and thus its function. We also hypothesized that changes in apoptotic and autophagic levels may be responsible for changes in the HG. To test these hypotheses, we studied the ultrastructural changes in the HG of hamsters under different daylight lengths. On this basis, the protein and mRNA levels of apoptosis (bax, bcl2, and Cyto C) and autophagy (LC3 and P62)-related indicators were studied. We then quantified mitochondrial function (ATP synthase and CS) and fission level (Drp1, Mff, and Fis1), and measured the synthesis of MT (AANAT and HIOMT) and SS.

## Materials and Methods

### Animals and Treatments

Striped dwarf hamsters were prepared as described previously in our laboratory (Xue et al., 2014; Zhao et al., 2014). In early March 2019, hamsters were captured from cropland in the Qufu region of Shandong Province, China (N35.78° E117.01°). This area experiences a temperate continental monsoon climate, with marked changes in seasonal light and temperature. The main crops in the area are wheat, peanuts, and corn.

The captured hamsters were acclimated in an animal feeding room and exposed to natural light for about two weeks. Hamsters were housed individually in cages (28 × 18 × 12 cm) at an ambient temperature of 22 ± 2°C and relative humidity of 55% ± 5%. Food (standard rat chow, Jinan Pengyue Experimental Animal Breeding Co., Ltd., China) and water were provided *ad libitum*. All procedures followed the Laboratory Animal Guidelines for the Ethical Review of Animal Welfare (GB/T 35892-2018) and were approved by the Animal Care and Use Committee of Qufu Normal University (Permit Number: dwsc2019010).

Based on body weight and degree of wear on upper molars, a total of 60 adult female hamsters (20–35 g) were randomly divided into three groups of 20 animals each. These groups then underwent long photoperiod (16:8 h light:dark cycle; light from 04:00 to 20:00, LP), moderate photoperiod (12:12 h light:dark cycle; light from 06:00 to 18:00, MP), or short photoperiod treatment (8:16 h light:dark cycle; light from 08:00 to 16:00, SP).

For photoperiod treatment, the hamsters were placed into a biodiverse small-animal feeding system (NK, LP-30LED-8CTAR, Osaka, Japan). The conditions of the system were 22 ± 2°C temperature, 55% ± 5% relative humidity, and 150 ± 10 lx light intensity. Photoperiod treatment began on 17 March, when the sun rose at 06:20 and set at 18:20 (Supplementary Data Sheet 1), and lasted 10 weeks. Every afternoon of the last week, vaginal smears were used to detect the estrous cycle of the females.

### Sample Preparation

Hamsters were sacrificed by CO_2_ asphyxiation at 22:00 on the last day, after all hamsters had been in darkness for at least 2 h. Blood samples were collected immediately from the heart after sacrifice, stored at 4°C for 30 min, and then centrifuged at 3000 rpm for 15 min at 4°C. Serum MT levels were estimated using an enzyme-linked immunosorbent assay (Shanghai Hengyuan Biological Technology Co., Ltd., H-40277, Shanghai, China). The HGs were removed, with lengths and weights then recorded. The HGs from five hamsters were fixed by immersion and processed for light and electron microscopy. The right HGs were immersed in 10% buffered formalin for paraffin section embedding, and the left HGs were immersed in glutaraldehyde-paraformaldehyde for transmission electron microscopy (TEM). The remaining HGs from 15 hamsters in each group were frozen in liquid nitrogen and stored in a refrigerator at −80°C for subsequent Western blotting, real-time polymerase chain reaction (RT-PCR), and immunofluorescence histochemical analyses. In each experiment, unilateral HGs from 10 hamsters were used for quantification. All procedures were carried out in accordance with approved guidelines.

### Histological Studies

Hematoxylin-eosin (HE) staining was performed to assess histological changes in HG cells. The HGs were embedded in paraffin blocks and serial sections (5 μm) were made through the entire gland. After rehydration, the sections were stained in hematoxylin dyeing solution for 30 min and slowly washed with running water for at least 15 min. Differential staining was performed using 1% hydrochloric acid-alcohol solution for 15 s, followed by slow rinsing with running water for at least 5 min. The slides were then stained with 1% eosin Y solution for 5 min and then dehydrated across an ethanol gradient, followed by xylene. One drop of neutral balsam mounting medium was placed on each slide and then covered with a coverslip. The mounted slides were observed using an optical microscope (Olympus, BX51, Tokyo, Japan).

### Transmission Electron Microscopy (TEM)

The HGs were cut into blocks and immersed in 3% glutaraldehyde-paraformaldehyde. The blocks were then dehydrated with a graded series of ethanol and embedded in epoxy resin, with TEM then performed as described previously (Wang et al., 2019). A semithin section was applied to tissue samples, and after methylene blue staining (Biazik et al., 2015), sections were adjusted under the microscope and sliced with an ultramicrotome (LKB-NOVA, United States). The ultrathin sections were double-stained with Reynolds’ lead citrate and ethanolic uranyl acetate (Reynolds, 1963) and then examined via TEM (JEOL, JEM-100SX, Japan). Images were processed with NIH Image software (Image-Pro Plus 6.0).

### Fluorescence Immunohistochemical Analysis

Frozen 10-μm thick tissue cross-sections were cut from the mid-belly (middle) of the two lobes of each HG sample at −20°C with a cryostat (Leica, CM1950, Germany) and then stored at −80°C for further staining. Ten sections from each lobe were randomly selected for follow-up experiments. After 15 min of immersion in distilled water, the sections were stained in blocking solution [5% bovine serum albumin (BSA)] for 30 min at room temperature and then incubated with rabbit anti-LC3 (1:200, #ab48394, Abcam, Cambridge, United Kingdom) or rabbit anti-P62 (1:200, #18420, Proteintech, Wuhan, China) solution at 4°C overnight. The sections were subsequently incubated with goat anti-rabbit Alexa Fluor 488 (1:200, #11034, Thermo Fisher Scientific, Rockford, IL, United States) at 37°C for 2 h, and then with anti-laminin rabbit polyclonal antibody (1:500, #BA1761, Boster, Wuhan, China) and goat anti-rabbit Alexa Fluor 647 (1:200, #21245, Thermo Fisher Scientific) under the same conditions. Finally, the sections were counterstained with 4′6′-diamidino-2-phenylindole (DAPI) (1:100, #D1306, Sigma-Aldrich, Saint Quentin Fallavier, France) at 37°C for 30 min. Images were visualized using a confocal laser scanning microscope (ZEISS, 880NLO, Germany) under krypton/argon laser illumination at 350, 488, and 647 nm emitted light, with capture at an emitting fluorescence of 461, 526, and 665 nm. Protein aggregations of LC3 and P62 were counted using a 100 μm × 100 μm area.

### Terminal Deoxynucleotidyl Transferase Biotin-dUTP Nick End Labeling (TUNEL) Staining

DNA fragmentation induced by apoptosis was determined by double-labeled fluorometric TUNEL detection as described previously (Fu et al., 2016). The frozen sections were permeabilized with 0.2% Triton X-100 in 0.1% sodium citrate at 4°C for 2 min and then incubated with an anti-laminin rabbit polyclonal antibody (1:500) at 4°C overnight. After washing with PBS for 30 min, the sections were incubated with fluorochrome-conjugated secondary AF647 antibodies at room temperature for 2 h. Subsequently, TUNEL (#MK1023, Boster) reaction mixture was added at the recommended 1:9 ratio, and the sections were incubated for 60 min at 37°C in a humidified chamber in the dark, per the manufacturer’s protocols. Finally, the sections were counterstained with DAPI. Imaging was performed using a confocal laser scanning microscope with the same excitation and emission wavelengths as described above.

### Western Blotting

Western blotting was conducted as described previously (Wang et al., 2019). Protein was extracted from HGs and solubilized in sample buffer (100 mM Tris pH 6.8, 5% 2-β-mercaptoethanol, 5% glycerol, 4% SDS and bromophenol blue), with protein extracts subsequently fractionated by SDS–PAGE using Laemmli gels and transferred to PVDF membranes (0.45-μm pore size) using a Bio-Rad semi-dry transfer apparatus. The blotted membranes were blocked with 1% BSA in Tris-buffered saline (TBS; 150 mM NaCl, 50 mM Tris–HCl, pH 7.5) and incubated with rabbit anti-bax (1:1000, #50599, Proteintech), rabbit anti-bcl2 (1:1000, #3498, Cell Signaling Technology CST, Danvers, MA, United States), rabbit anti-Cyto C (1:1000, #11940, CST), rabbit anti-LC3 (1:1000), rabbit anti-P62 (1:1000), rabbit anti-AANAT (1:1000, #17990, Proteintech), rabbit anti-HIOMT (1:1000, ab180511, Abcam, Cambridge, United Kingdom), rabbit anti-MTR (1:1000, ab87639, Abcam), rabbit anti-somatostatin (1:1000, DF13243, Affinity Biosciences, OH, United States), rabbit anti-ATP synthase (1:1000, #14676, Proteintech), rabbit anti-citrate synthase (1:1000, #16131, Proteintech), rabbit anti-Drp1 (1:1000, #12957, Proteintech), rabbit anti-Mff (1:1000, #17090, Proteintech), rabbit anti-Fis1 (1:1000, #10956, Proteintech), and rabbit anti-β-actin (1:5000, #20536, Proteintech) in TBS containing 0.1% BSA at 4°C overnight. The membranes were then incubated with IRDye 800 CW goat-anti rabbit secondary antibodies (1:5000, #31460, Thermo Fisher Scientific) for 90 min at room temperature and visualized with an Odyssey scanner (Bio-Rad, Hercules, CA, United States). Quantification analysis of the blots was performed using NIH Image J software.

### Quantitative RT-PCR (qRT-PCR)

Total RNA was routinely extracted from muscles using an RNAiso Plus kit (TaKaRa, Dalian, China) according to the manufacturer’s protocols. RNA quality was determined by measuring the OD260/OD280 ratio, with samples showing a ratio of > 1.8 then reverse transcribed into cDNA using a TaKaRa reagent and stored at −80°C for subsequent reactions. qRT-PCR was performed using a SYBR Premix Ex Taq II kit (TaKaRa). Dissolution and amplification curves were first observed, with the right curve then chosen. The reference gene was β*-*actin and the 2^–^^Δ^^Δ^^*ct*^ method was used to analyze the relative concentrations of *bax*, *bcl2*, *lc3*, *p62*, and *ss* mRNA. The primers used for qRT-PCR (Sangon, Shanghai, China) are shown in Table 1.

**TABLE 1:** Primer sequences and related information.

	**Primer sequences**	**Annealing**	**Length of**
**Primers**	**(5′-3′)**	**temperature**	**products**
*bax*	F: GCGAATTGGAGATGAGCTGGACAG	59°C	124 bp
	R: TGCCACACGGAAGAAGACCTCTC		
*bcl2*	F: TACGGTGGTGGAGGAACTCTTCAG	59°C	169 bp
	R: GGTGTGCAGATGCCGGTTCAG		
*lc3*	F: TCGCCGACCGCTGTAAGGAG	59°C	169 bp
	R: CGCCGGATGATCTTGACCAACTC		
*p62*	F: AGGAGGAGACGATGACTGGACAC	59°C	150 bp
	R: TTGGTCTGTAGGAGCCTGGTGAG		
*ss*	F: AGCGGCTGAAGGAGACG	59°C	170 bp
	R: GGGTTTGGGGGAGAGG		
β*-actin*	F: GAGACCTTCAACACCCCAGC	59°C	263 bp
	R: ATGTCACGCACGATTTCCC		

### Statistical Analyses

The normality of data and homogeneity of variance were tested by Shapiro-Wilk and Levene, respectively. Single factor analysis of variance (one-way ANOVA) was used to compare differences between groups. Fisher’s least significant difference (LSD) *post hoc* test or Dunnett’s T3 method were used to determine group differences. The differences were considered significant when *P* < 0.05, highly significant when *P* < 0.01, and very highly significant when *P* < 0.001. Data are expressed as means ± standard deviation (Mean ± SD). All statistical analyzes were conducted using SPSS 19.0.

## Results

### Hamster Body Weight (BW), Carcass Weight (CW), HG Weight (HGW) and HGW to CW Ratio (HGW/CW)

No significant differences in BW were observed among the three groups before the experiment. After two months of photoperiod treatment, however, the BW values in the SP and LP groups were lower (5%, *P* < 0.05) than that in the MP group. In addition, CW was lower (*P* < 0.05) in the SP and LP groups than that in the MP group. Furthermore, HGW was significantly lower in the SP (10%, *P* < 0.05) and LP (9%, *P* < 0.05) groups than that in the MP group, although the HGW/CW ratio demonstrated no significant differences among the three groups (Table 2).

**TABLE 2:** Effects of photoperiod on body weight (BW), carcass weight (CW), Harderian gland weight (HGW) and ratio of HGW/CW in hamsters after 10 weeks.

**Group**	**SP**	**MP**	**LP**
BW before photoperiod (g)	20.98 ± 1.15	21.06 ± 1.26	20.99 ± 1.21
BW after photoperiod (g)	20.63 ± 0.89	21.80 ± 1.36*	20.61 ± 1.40^#^
CW after photoperiod (g)	15.20 ± 1.45	16.26 ± 1.85*	14.80 ± 1.91^#^
HGW after photoperiod (mg)	24.48 ± 2.54	27.13 ± 2.72*	24.66 ± 2.27^#^
HGW/CW after photoperiod (mg/g)	1.60 ± 0.13	1.67 ± 0.13	1.67 ± 0.17

SP, short photoperiod; MP, moderate photoperiod; LP, long photoperiod. Data represent mean ± SD; *n* = 20. *P < 0.05 compared with SP, ^#^*P* < 0.05 compared with MP.

### Blood MT Levels Under Different Photoperiods

**FIGURE 1:**
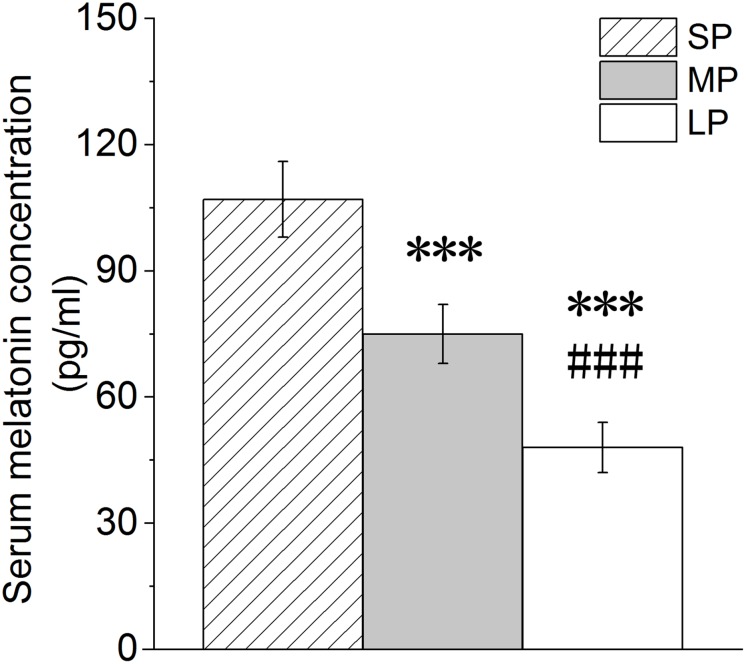
Changes in melatonin level in blood of hamsters in three photoperiodic groups. Values are means ± SD. *n* = 20. SP, short photoperiod; MP, moderate photoperiod; LP, long photoperiod.^*⁣**^P < 0.001 compared with SP; ^###^*P* < 0.001 compared with MP.

Melatonin receptors exist in a variety of organs and tissue cells. Thus, MT may be involved in the mediation of photoperiod action on physiological changes in organisms (Bubenik et al., 1976). Here, the level of serum MT decreased with the increase in illumination duration, with the serum MT level in the MP and LP groups found to be significantly lower than that in the SP group (Figure 1).

### External and Histological Morphology of HG

**FIGURE 2:**
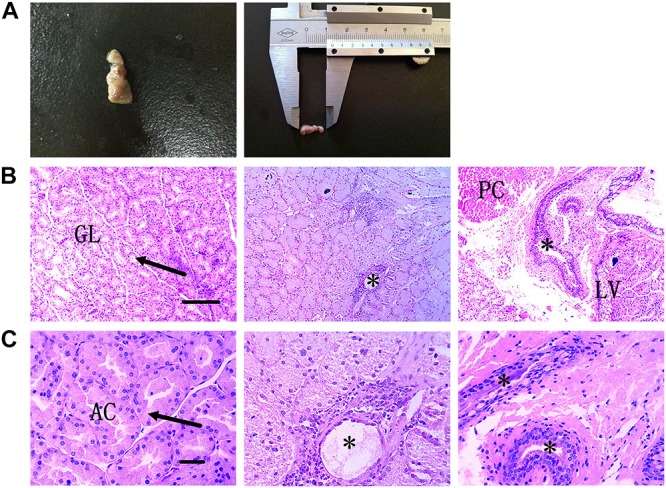
External anatomical structure and histological structure of HG in hamsters. **(A)** Morphological structure of HG. **(B)** Plasma cells and secretory ducts in HG are shown under low-power magnification. Scale bar = 100 μm. **(C)** Plasma cells and secretory ducts in HG are shown under high-power magnification. Scale bar = 20 μm. Arrow, acinar cell; asterisk, secretory duct; PC, plasma cells; LV, lipid vacuolation; GL, gland leaflet; AC, acinar cavity.

The flat dumbbell-shaped HG is located in the eye socket at the back of the eyeball. It is divided into large and small lobes and is enclosed in the posterior part of the eyeball where it connects with the temporal muscle. It has a smooth outline and is covered with a connective tissue capsule. The HG is mainly pink to dark red, with slightly different colors between the large and small leaves. Here, the lengths and weights of adult HGs were ∼0.9–1.2 cm and 0.02–0.03 g, respectively (Figure 2A).

The hamster HG is a complex alveolate structure. Each gland lobe is divided into many lobules by connective tissue and is composed of various acini and ducts. The acinar epithelium is composed of a layer of columnar glandular epithelial cells. The cylindrical or conical epithelial cells form tubular structures and many acinar ducts. The nucleus is round or oval and the subepithelial basement membrane contains many plasma cells and several lymphocytes (Figures 2B,C).

### Ultrastructural Changes in HG Nuclei, Mitochondria, and Autophagolysosomes

**FIGURE 3:**
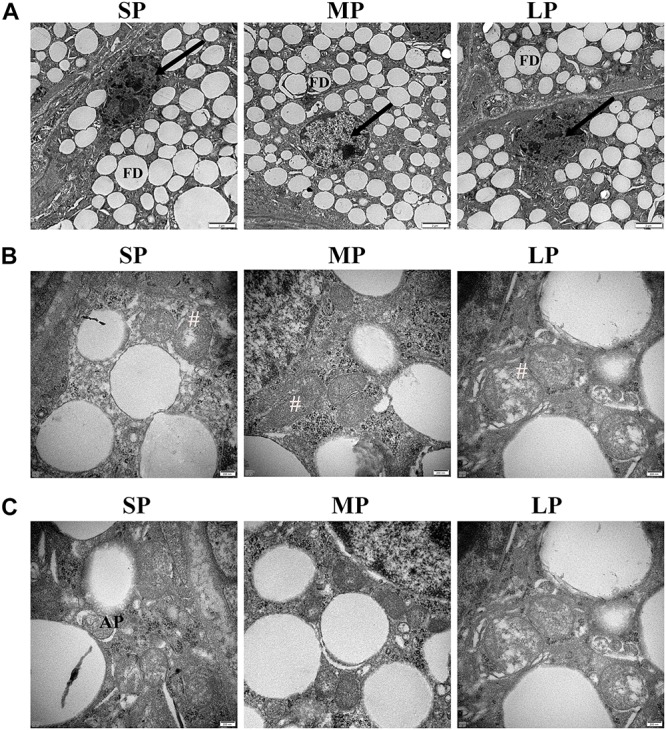
Ultrastructure of HG in hamsters from three photoperiodic groups. **(A)** Nucleus ultrastructure of HG in hamsters from three photoperiodic groups. In SP and LP groups of hamsters, nuclei of secreting cells were deformed, and chromatin were condensed. Nuclei of mesenchymal cells were irregular, and chromatin was obviously agglutinated. Large number of fat droplets (FD) were observed in secretory cells (see arrow) of HG, and plasma membrane (see asterisk) was smooth and clear. Scale bar = 2 μm. **(B)** Cristae of mitochondria of HG in hamsters from three photoperiodic groups. In SP and LP groups, mitochondria (#) of secretory cells were swollen and cristae were disordered. Mitochondria in LP group were slightly swollen. In MP group, mitochondria were well formed, and cristae were obvious. Scale bar = 0.2 μm. **(C)** Autophagolysosomes of HG in hamsters from three photoperiodic groups. Significant autophagolysosomal structures (AP) were observed in SP group. In other groups, autophagolysosomal structures were hardly observed. Scale bar = 0.2 μm. SP, short photoperiod; MP, moderate photoperiod; LP, long photoperiod.

We observed many secretory cells in the HGs of the three different photoperiod groups in females, including many round- or elliptical-shaped fat droplets. The plasma membrane of the secretory cells was clearly visible. The nuclei of the HG secretory cells were obviously deformed in the SP group, with chromatin condensation also observed in these cells in the LP group. In contrast, chromatin concentration was rarely observed in the HGs of the MP group, which may be related to the level of apoptosis (Figure 3A).

In the MP group, the mitochondria in the HG were irregularly oval, the cristae were clearly visible, and the membrane was complete. In the LP group, mitochondrial swelling, cristae rupture, and membrane disintegration were observed. In the SP group, the mitochondria were round and slightly swollen, and the cristae were fractured (Figure 3B).

Typical autophagolysosomal structures were observed in the SP group, showing a clear membrane structure on the outside and wrapped contents in the middle. In the other groups, however, typical autophagolysosomal structures were difficult to observe (Figure 3C).

### DNA Fragmentation

**FIGURE 4:**
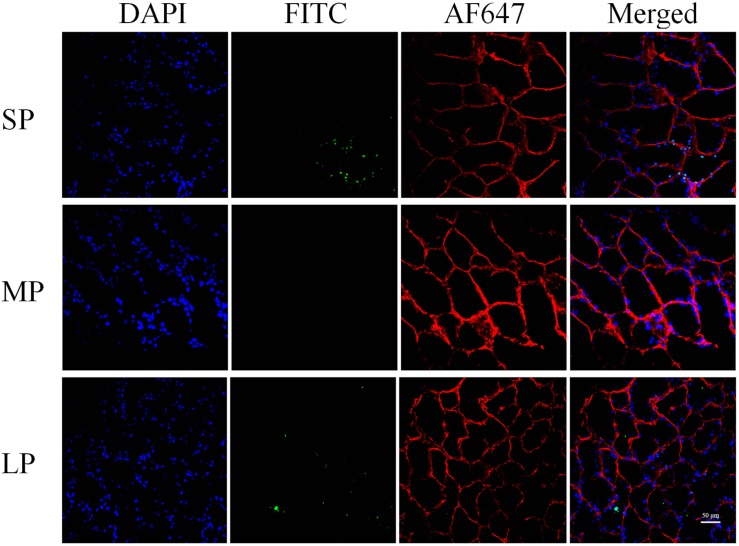
Fluorescent terminal deoxynucleotidyl transferase biotin-dUTP nick end labeling (TUNEL) staining of HG in hamsters in three photoperiodic groups. Immunofluorescence histochemistry showing cell apoptosis, cell boundaries, and nuclei. Blue represents 4′6′-diamidino-2-phenylindole (DAPI)-stained nucleus, red represents Alexa Fluor 647-stained laminin of interstitial tissue, green represents TUNEL by FITC. Scale bar = 50 μm. SP, short photoperiod; MP, moderate photoperiod; LP, long photoperiod.

TUNEL staining provided direct evidence of apoptosis. In the MP group, random HG sections showed that DNA fragmentation (represented by green fluorescence) was hardly observed. In the SP and LP groups, however, green fluorescence was observed in several adjacent cells and coincided with blue fluorescence in the nucleus, indicating increased apoptosis in both groups (Figure 4).

### Changes in LC3 and P62 Puncta in HG Under Different Photoperiods

**FIGURE 5:**
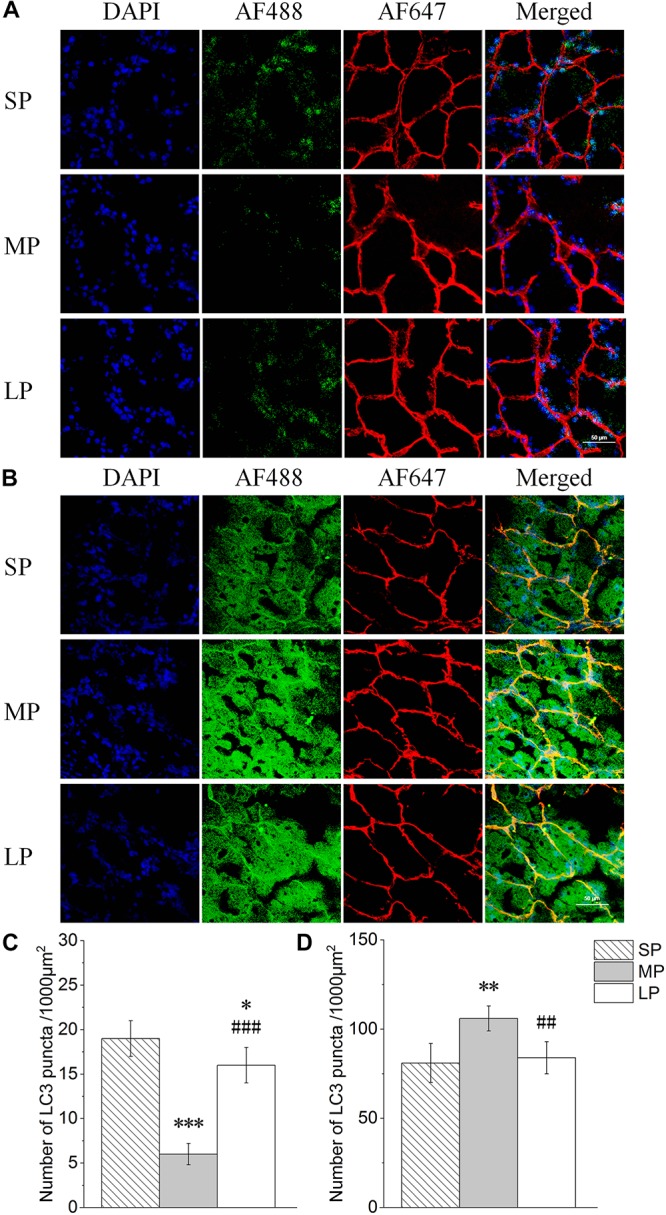
Quantification of LC3 and P62 puncta of HG in hamsters in three photoperiodic groups. **(A)** Immunofluorescence histochemistry showing LC3 puncta. Blue represents 4′6′-diamidino-2-phenylindole (DAPI)-stained nucleus, red represents Alexa Fluor 647-stained laminin of interstitial tissue, green represents AF488-stained LC3. Scale bar = 50 μm. **(B)** Immunofluorescence histochemistry showing P62 puncta. Blue represents 4′6′-diamidino-2-phenylindole (DAPI)-stained nucleus, red represents Alexa Fluor 647-stained laminin of interstitial tissue, green represents AF488-stained P62. Scale bar = 50 μm. **(C)** Quantification of LC3 puncta. **(D)** Quantification of P62 puncta. Six figures were analyzed in each sample; ten samples were analyzed in each group. Values are means ± SD. SP, short photoperiod; MP, moderate photoperiod; LP, long photoperiod.^∗^*P* < 0.05, ^∗∗^*P* < 0.01, ^∗∗∗^*P* < 0.001 compared with SP; ^##^*P* < 0.01,^###^*P* < 0.001 compared with MP.

The number of cytoplasmic LC3 puncta per 1000 μm2 is indicative of LC3I to LC3II conversion. Representative LC3 immunofluorescence staining is shown in Figure 5A. Results demonstrated significantly higher cytoplasmic LC3 puncta in the SP and LP groups (230% and 182%, respectively; *P* < 0.001) than that in the MP group (Figure 5C).

Representative P62 immunofluorescence staining is shown in Figure 5B. The number of P62 puncta was highest in the MP group, with a 27% increase compared to that in the LP group (*P* < 0.01) (Figure 5D).

### Relative Protein Expression

**FIGURE 6:**
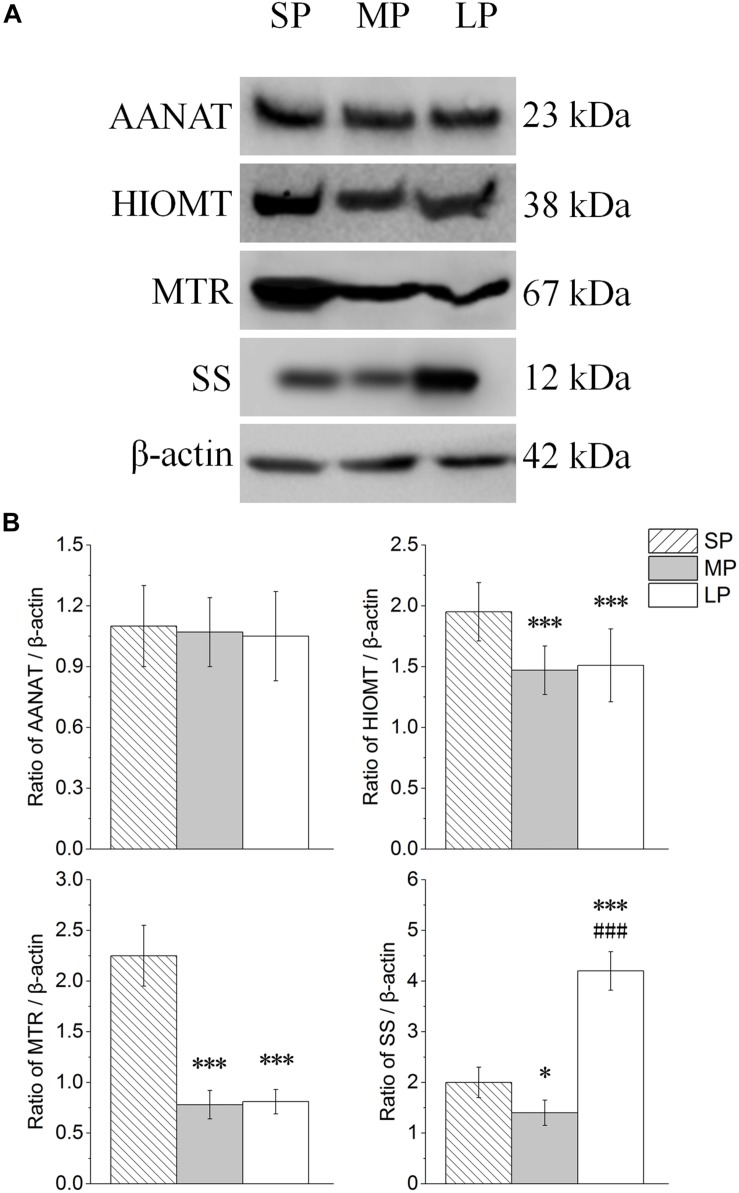
Changes in protein levels of AANAT, HIOMT, MTR, and SS in HG of hamsters in three different photoperiodic groups. **(A)** Representative immunoblots of AANAT, HIOMT, MTR, SS, and β-actin in three different photoperiodic groups. **(B)** Ratio of AANAT, HIOMT, MTR, SS to β-actin in HG of hamsters in three different photoperiodic groups. Values are means ± SD. *n* = 10. SP, short photoperiod; MP, moderate photoperiod; LP, long photoperiod. ^∗^*P* < 0.05, ^∗∗∗^*P* < 0.001 compared with SP; ^###^*P* < 0.001 compared with MP.

The contents of AANAT, HIOMT, MTR, and SS were detected by Western blot analysis, as shown in Figure 6A. The protein expression levels of AANAT showed no significant differences among the three groups. HIOMT and MTR both showed highest protein expression in the SP group (*P* < 0.01). Among the groups, SS expression was lowest in the MP group (Figure 6B).

**FIGURE 7:**
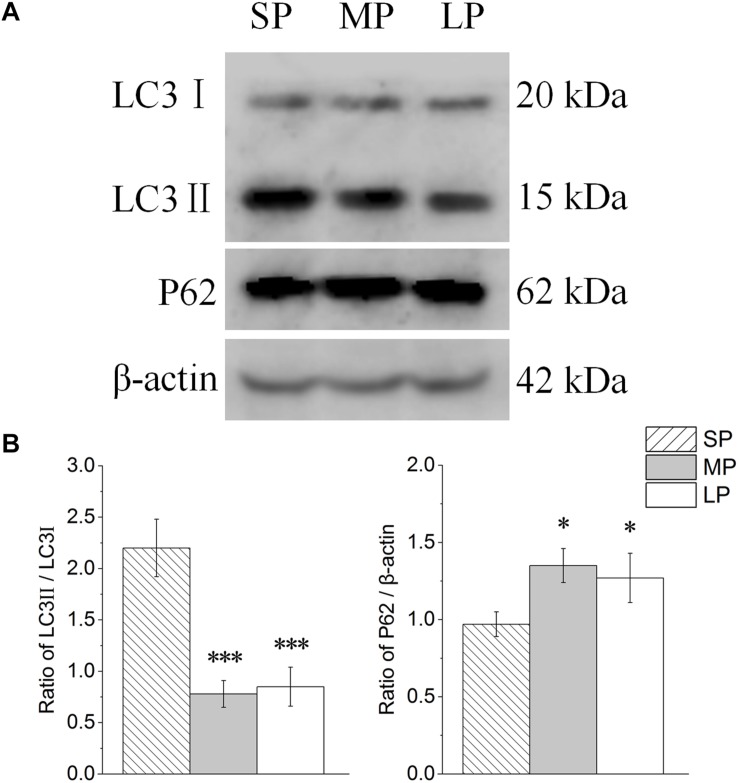
Changes in protein levels of LC3 and P62 in HG of hamsters in three different photoperiodic groups. **(A)** Representative immunoblots of LC3, P62, and β-actin in three different photoperiodic groups. **(B)** Ratio of LC3, P62 to β-actin in HG of hamsters in three different photoperiodic groups. Values are means ± SD. *n* = 10. SP, short photoperiod; MP, moderate photoperiod; LP, long photoperiod. ^∗^*P* < 0.05, ^∗∗∗^*P* < 0.001 compared with SP; ^#^*P* < 0.05, ^###^*P* < 0.001 compared with MP.

The contents of LC3 and P62 were detected by Western blot analysis, as shown in Figure 7A. The LC3II/LC3I level was lower in the LP group than that in the SP and MP groups (*P* < 0.001). The protein expression of P62 showed the opposite trend to that of LC3II/LC3I (Figure 7B).

**FIGURE 8:**
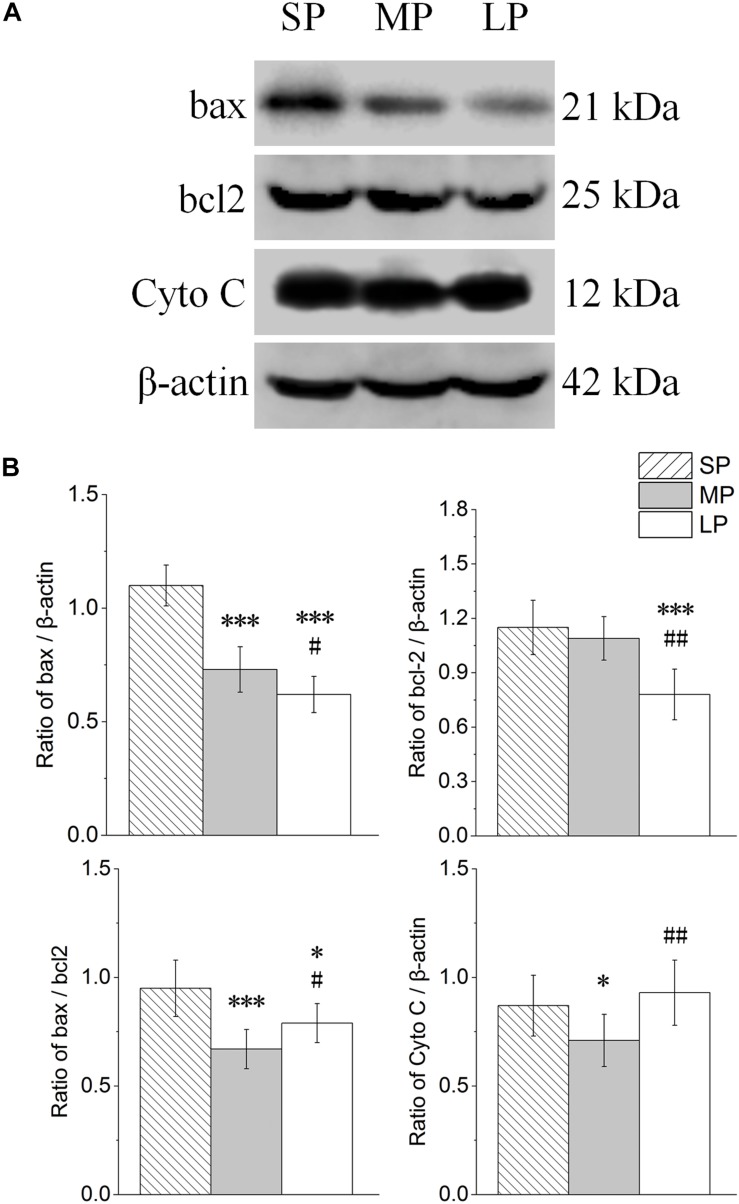
Changes in protein levels of bax, bcl2, Cyto C, and bax/bcl2 in HG of hamsters in three different photoperiodic groups. **(A)** Representative immunoblots of bax, bcl2, Cyto C, and β-actin in three different photoperiodic groups. **(B)** Ratio of bax, bcl2, Cyto C to β-actin and ratio of bax to bcl2 in HG of hamsters in three different photoperiodic groups. Values are means ± SD. *n* = 10. SP, short photoperiod; MP, moderate photoperiod; LP, long photoperiod. ^∗^*P* < 0.05, ^∗∗∗^*P* < 0.001 compared with SP; ^#^*P* < 0.05, ^##^*P* < 0.01 compared with MP.

The contents of bax, bcl2, and Cyto C were detected by Western blot analysis, as shown in Figure 8A. The ratio of bax/bcl2 and protein expression of Cyto C were lowest in the MP group among the three groups (Figure 8B).

**FIGURE 9:**
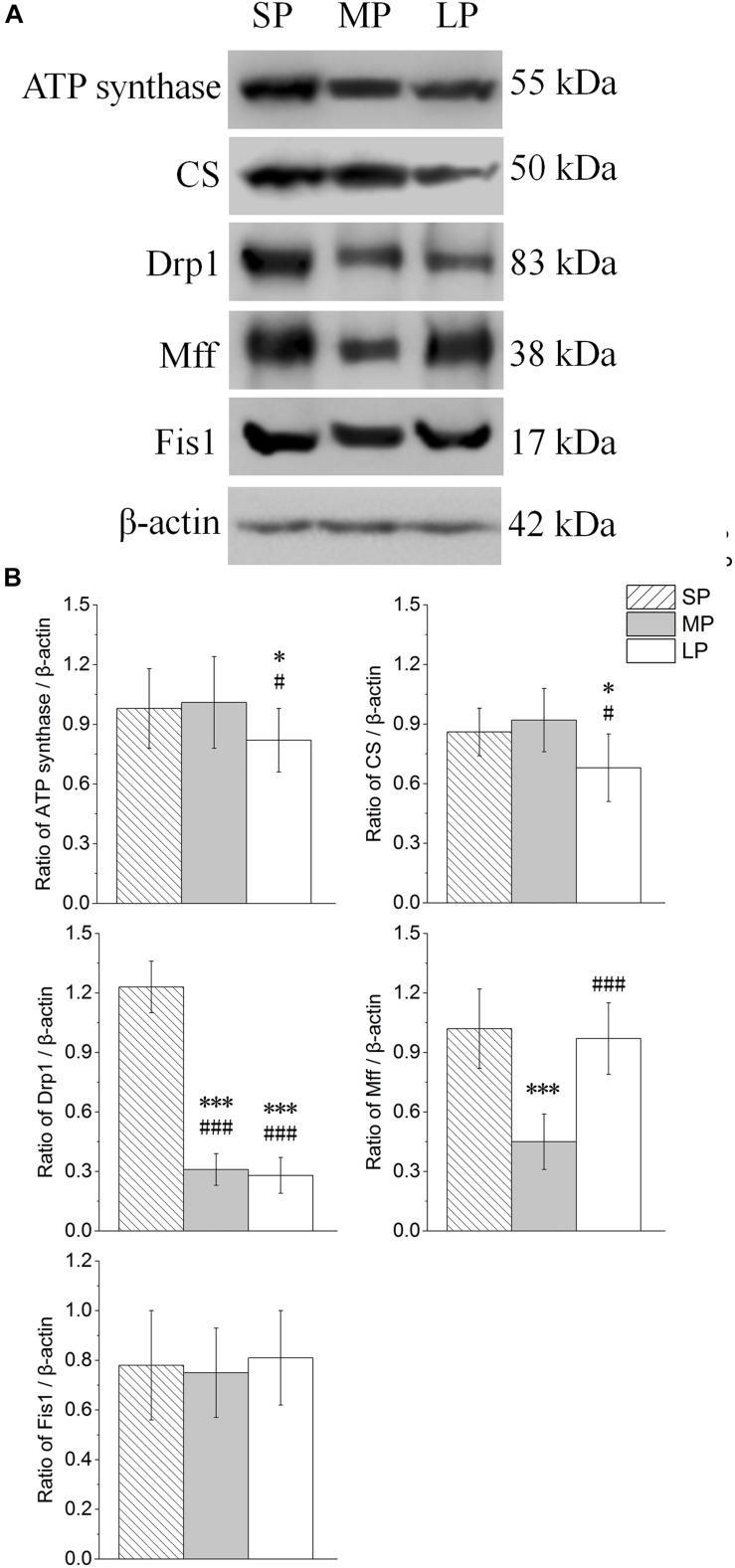
Changes in protein levels of ATP synthase, CS, Drp1, Mff, and Fis1 in HG of hamsters in three different photoperiodic groups. **(A)** Representative immunoblots of ATP synthase, CS, Drp1, Mff, Fis1, and β-actin in three different photoperiodic groups. **(B)** Ratio of ATP synthase, CS, Drp1, Mff, Fis1 to β-actin in HG of hamsters in three different photoperiodic groups. Values are means ± SD. *n* = 10. SP, short photoperiod; MP, moderate photoperiod; LP, long photoperiod. ^∗^*P* < 0.05, ^∗∗∗^*P* < 0.001 compared with SP; ^#^*P* < 0.05, ^###^*P* < 0.001 compared with MP.

The contents of ATP synthase, CS, Drp1, Mff, and Fis1 were detected by Western blot analysis, as shown in Figure 9A. The ATP synthase protein expression level was lowest in the LP group among the three groups, and the protein expression of CS showed the same trend (Figure 9B).

The Drp1 and Mff protein expression levels were highest in the SP group among the three groups. Inhibited protein expression was not significantly different among the three groups (Figure 9B).

### Relative mRNA Expression

**FIGURE 10:**
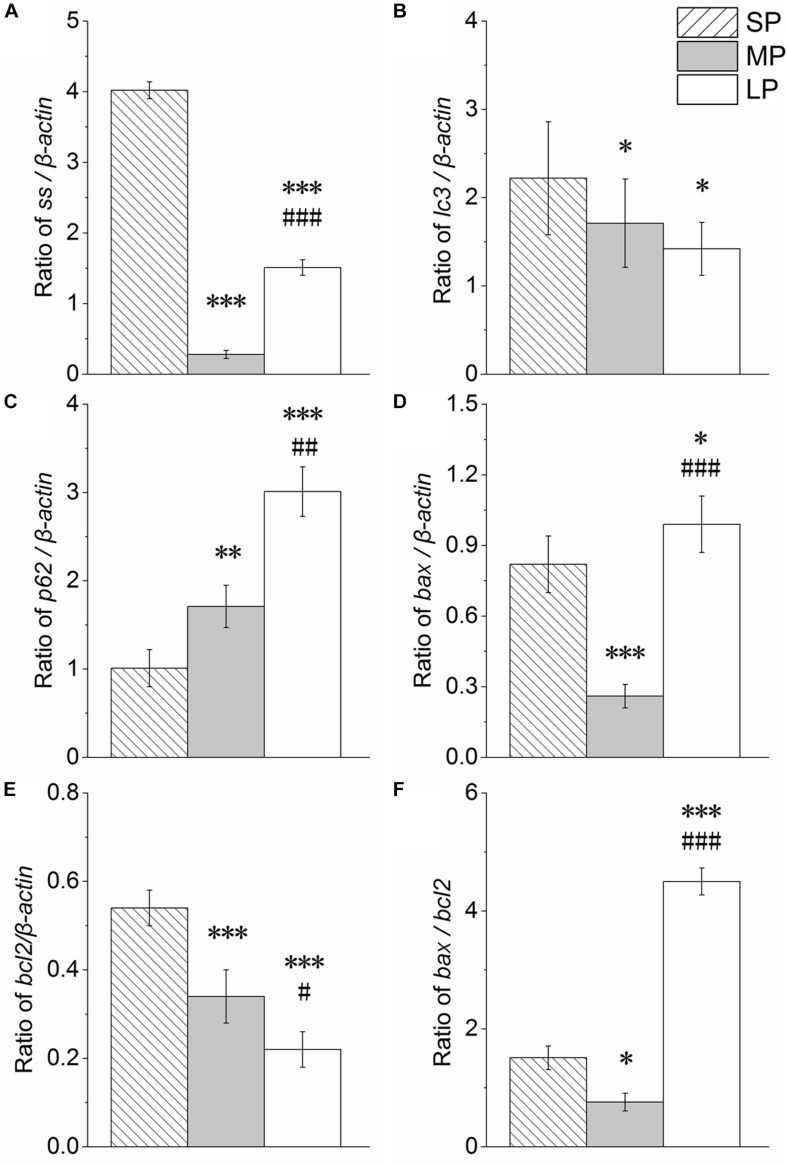
Changes in mRNA levels of *ss, lc3, p62, bax, bcl2*, and *bax/bcl2* in HG of hamsters in three different photoperiodic groups. **(A)** Ratio of *ss* to β*-actin*. **(B)** Ratio of *lc3* to β*-actin*. **(C)** Ratio of *p62* to β*-actin*. **(D)** Ratio of *bax* to β*-actin*. **(E)** Ratio of *bcl2* to β*-actin*. **(F)** Ratio of *bax* to *bcl2*. Values are means ± SD. *n* = 10. SP, short photoperiod; MP, moderate photoperiod; LP, long photoperiod. ^∗^*P* < 0.05, ^∗∗^*P* < 0.01, ^∗∗∗^*P* < 0.001 compared with SP; ^#^*P* < 0.05, ^##^*P* < 0.01, ^###^*P* < 0.001 compared with MP.

Our results showed that *ss* expression was higher in the SP and LP groups (*P* < 0.001) than that in the MP group (Figure 10A).

The *lc3* and *p62* mRNA expression levels are shown in Figures 10B,C, respectively. The *lc3* mRNA expression levels followed the order SP > MP > LP (*P* < 0.05). The *p62* mRNA expression levels followed the order LP > MP > SP (*P* < 0.05).

The *bax* and *bcl2* mRNA expression levels are shown in Figures 10D,E, respectively. *Bax* expression (*P* < 0.001) and *Bcl2* expression (*P* < 0.05) were both higher in the SP and LP groups than that in the MP group.

The *bax/bcl2* ratio, which is an important indicator of apoptosis, was analyzed and is shown in Figure 10F. The ratio was lower in the SP and LP groups (*P* < 0.05) than that in the MP group.

## Discussion

**FIGURE 11:**
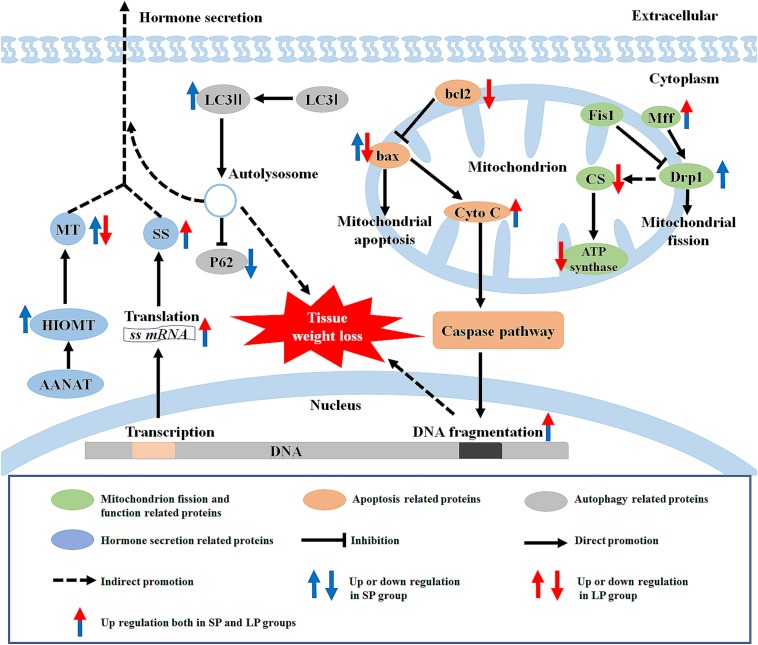
Graphical summary of study. LC3, microtubule-associated protein 1 light chain; MT, melatonin; HIOMT, hydroxyindole-O-methyltransferase; AANAT, arylalkylamine-N-acetyltransferase; SS, somatostatin; P62, sequestosome 1; Cyto C, cytochrome C; Fis1, fission 1; Mff, mitochondrial fission factor; Drp1, dynamin-related protein 1; ATP synthase, adenosine triphosphate synthase; CS, citrate synthase; SP, short photoperiod; LP, long photoperiod.

Our results showed that, compared with the control (moderate photoperiod) group, the body weight, carcass weight, and HG weight of hamsters were significantly reduced under short and long photoperiods. The protein expression levels of SS were significantly higher under the short and long photoperiods than that under the moderate photoperiod. The autophagy level increased under the short photoperiod, and the apoptosis level increased under both short and long photoperiod conditions. In addition, the protein expression levels of mitochondrial ATP synthase, CS, and Drp1 were reduced in the HG of hamsters under long photoperiod conditions, suggesting that some mitochondrial functions of the HG may have been weakened at this time (Figure 11).

### Photoperiod Changes in Morphology and Hormone Synthesis in HG

After 10 weeks of different light treatment, the body and carcass weights of the striped dwarf hamsters were significantly lower in the short and long photoperiod groups than that before treatment or in the control (moderate photoperiod) group. This result is somewhat similar to that found in Siberian and European hamsters (*Cricetus cricetus*), i.e., where SP caused weight loss (Bartness and Wade, 1985; Bertoni et al., 1992; Masson-Pevet et al., 1994; Mauer and Bartness, 1994). As there were no significant differences in the HG weight to carcass weight ratios among the three groups, and because other species such as Siberian and golden hamsters have shown that weight loss caused by photoperiod is accompanied by loss of visceral organ mass (Reiter, 1975; Bertoni et al., 1992), which suggested the loss of weight in the SP and LP groups may be related to the loss of body tissue weight in this study. At the same time, striped dwarf hamster body weight also decreased under long photoperiod conditions, which may be because summer (i.e., long photoperiod) is a non-breeding season, and weight reduction is conducive to energy conservation. This is similar to previous study, which reported that the body weights of striped dwarf hamsters tend to remain at the same level after 54 d of short and long photoperiod treatment (Xu and Hu, 2017). In addition, Siberian hamsters become insensitive to light under short photoperiod of more than 14 weeks (Rendon et al., 2017). In this study, the similarity of short and long photoperiod after 8 weeks photoperiod treatment may also be related to the sensitivity of animals to light, which needs to be further verified. Similarly, the HG weight in the short and long photoperiod groups was lower than that in the moderate photoperiod group. However, the HG weight to carcass weight ratio in the short and long photoperiod groups was not lower, suggesting that the decrease in HG weight was consistent with a decrease in animal body weight.

Here, HE staining showed that the HG contained a large number of secretory chambers, indicating a possible secretory function, as seen in previous studies on rats, sheep, and other mammals (Leshin and Jackson, 1987; Jimenez-Jorge et al., 2007). Similarly, ultrastructural observation of the HG also identified many lipid droplets in the cells, again indicating that the HG may have a secretory function.

Our results also showed that the concentration of MT in the blood and protein expression of HIOMT in the HG were highest under short photoperiod conditions, whereas AANAT remained unchanged in the three groups. In animals, MT is the main hormone reflecting changes in the duration of the dark phase in the external environment, and its secretion is highest at night (Hartmann and Kluge, 1989; Arushanian and Arushanian, 1991). Our results showed that MT synthesis was positively correlated with the time hamsters entered darkness (6 h in short photoperiod, 4 h in moderate photoperiod, and 2 h in long photoperiod). That is, similar to the pineal gland, the HG participated in the regulation of photoperiod rhythm of body MT levels. It worth noting that there was no correlation between serum MT trends and body weight in the three groups. In our previous study, hamsters were injected with MT and compared with those in the SP group; however, the trend of gene changes related to reproduction in the hypothalamus was not similar to that in the photoperiod group (Li et al., 2015). We speculate that the influence of light in an animal’s body is not only reflected by changes in MT level, but also related to other factors such as neuroregulation. Cells with MTR can sense the length of day and night through changes in MT levels, thus affecting signal molecules in the cells (Reiter, 1980; Yasuo et al., 2009). In this study, the high expression of MTR in the short photoperiod group suggests that it may be more sensitive to changes in MT levels. In addition, high SS protein and mRNA levels in the HG were found under short and long photoperiod conditions. This is similar to that reported in rats, i.e., expression of SS in the HG is significantly higher in winter than in spring and summer (Mato et al., 1996). Considering that SS is the main endogenous hormone for animal growth, this suggests that the HG may be involved in body weight loss during the non-breeding season photoperiod.

### Photoperiod Changes in Autophagy and Apoptosis Levels in HG

To explore the mechanism of the above phenomenon, we studied the autophagy level in the HG of hamsters under different photoperiods. We observed the formation of autophagolysosomes in the HG under short photoperiod conditions, suggesting that autophagy may occur in the HG. The most direct criterion for the occurrence of autophagy is whether the bilayer membrane vesicles enclosing substances or organelles can be observed under electron microscopy (Biazik et al., 2015; Dong et al., 2015). In this study, an autophagolysosomal structure encapsulated by a monolayer membrane was observed in the HG, indicating the possible occurrence of autophagy at this time. LC3II is a key protein of autophagolysosome membrane formation (Lee and Lee, 2016; Schaaf et al., 2016). Here, immunofluorescence histochemistry showed that LC3 protein aggregates, LC3II/LC3I protein expression, and LC3 mRNA expression were highest in the HG under short photoperiod conditions, proving that the autophagy level indeed increased from three dimensions. P62 is an autophagic transport protein, the accumulation of which indicates a decrease in autophagic efficiency (Lamark et al., 2017). The protein and mRNA expression levels of P62 were significantly lower in the female HG under short photoperiod conditions compared with levels in the other two groups, indicating an increase in autophagic efficiency. Therefore, at the protein and transcriptional level, the up-regulation of LC3 and down-regulation of P62 implied that autophagy in HG cells was significantly higher under short photoperiod conditions than under moderate photoperiod conditions. This may be one of the reasons for the decrease in HG weight in hamsters as this time. In mice, the secretory functions of the HG are associated with changes in autophagy levels (Koenig et al., 2015). Thus, we speculate that the increase in autophagy may contribute to the hormone secretion function of the HG.

Interestingly, ultrastructure analysis also showed significant chromatin agglutination and nuclear deformation in the secretory cells of the HG under short and long photoperiod conditions, suggesting that the secretory cells of the HG may have experienced apoptosis. The bax/bcl2 ratio is often used to measure the degree of cell apoptosis (Korsmeyer et al., 1993; Antonsson et al., 1997). High-intensity light stimulation or high-dose MT injection can lead to increased cell necrosis in the HG of female Syrian hamsters and male rats (Kurisu et al., 1996; Vega-Naredo et al., 2012). However, in other peripheral organs, such as the testes, short illumination can promote the expression of apoptotic markers such as bax, thus leading to organ atrophy (Morales et al., 2007; Shannonhouse et al., 2014). The results reported in this study are similar to the above research and showed that the bax/bcl2 ratio in the HG was significantly higher at both the protein and transcriptional level under short and long photoperiod exposure than that under moderate photoperiod conditions. The protein expression of Cyto C showed a similar trend, implying the up-regulation of apoptosis, which may be one of the main reasons for the decrease in HG weight in both groups under different photoperiods.

### Photoperiod Changes in Mitochondrial Function and Fission in HG

We also found that ATP synthase and CS protein expression levels were lower following long photoperiod treatment, consistent with the change in mitochondrial apoptosis level. CS is a rate-limiting enzyme in the tricarboxylic acid cycle and represents the ability of mitochondria to undertake aerobic oxidation (Wiegand and Remington, 1986; Remington, 1992). ATP synthase is the last step in ATP production by mitochondria, representing the ability of mitochondria to supply energy (Kramarova et al., 2008). In this study, the protein expression levels of both ATP synthase and CS in the HG were lower under long photoperiod conditions, indicating that mitochondrial function was weakened. In view of the obvious mitochondrial swelling in the long photoperiod group, we doubt whether it is related to the balance between mitochondrial apoptosis and fission in different photoperiods.

Dynamin-related protein 1 is a key factor related to the promotion of mitochondrial fission, with Mff and Fis1 found to up- and down-regulate Drp1 activity, respectively (Tieu and Nunnari, 2000; Yu et al., 2019). In the short photoperiod group, Drp1 protein expression increased by several times, whereas Mff increased significantly and Fis1 remained unchanged. This indicates that the mitochondrial fission level increased significantly. Based on this, and the increase in mitochondrial apoptosis, short photoperiod conditions may promote the regeneration of mitochondria in the HG of hamsters, which may be one of the reasons for the maintenance of mitochondrial function. Under long photoperiod conditions, Drp1, as the most important factor in mitochondrial fission, remained unchanged, although the expression of the Mff protein increased significantly, indicating that mitochondrial fission ability may not be enhanced significantly. The increase in mitochondrial apoptotic level and unchanged mitochondrial fission level may be the mechanisms for the decreased expression of ATP synthase and CS in mitochondria under long photoperiod conditions.

### Summary

In summary, we extend novel findings on the effects of photoperiod on morphological and functional changes in the HG and related mechanisms under different photoperiods. Melatonin synthesis function in the HG of hamsters was positively correlated with the time of entering darkness. The high level of SS secretion in the HG under short and long photoperiods compared to the moderate photoperiod control group may be involved in the observed weight loss of hamsters. We also demonstrated that the HG weight loss in hamsters under short and long photoperiod conditions may be due to the enhancement of autophagy under short photoperiod and apoptosis under both conditions. Mitochondrial function weakened under the long photoperiod treatment, which may be caused by maintenance of mitochondrial fission and up-regulation of apoptosis. Photoperiodic treatment during the non-breeding season led to different levels of degeneration in the morphology and function of the HG in hamsters, with the possible mechanism involving an imbalance of autophagy and apoptosis.

## Data Availability Statement

The datasets used and/or analyzed during the current study are available from the corresponding author on reasonable request. Partial original images are included in the supplementary documents.

## Ethics Statement

The animal study was reviewed and approved by the Animal Care and Use Committee of Qufu Normal University (Permit Number: dwsc2019010). All procedures followed the Laboratory Animal Guidelines for the Ethical Review of Animal Welfare (GB/T 35892-2018).

## Author Contributions

ZW, J-HX, and L-XX conceived and designed research. ZW, J-HX, J-JM, and X-TK performed experiments. ZW analyzed data and interpreted experimental results. ZW and J-JM prepared figures. ZW and J-HX drafted manuscript. MW and H-LX provided experimental guidance and suggestions for revision. J-HX, ZW, and L-XX edited and approved final version of manuscript.

## Conflict of Interest

The authors declare that the research was conducted in the absence of any commercial or financial relationships that could be construed as a potential conflict of interest.
