# Supplementary material for: Photoperiod Affects Harderian Gland Morphology and Secretion in Female Cricetulus barabensis: Autophagy, Apoptosis, and Mitochondria
Source: Front Physiol. 2020 May 6;11:408. doi: 10.3389/fphys.2020.00408 (PMC7218128; doi:10.3389/fphys.2020.00408)

Original data of sunrise and sunset time in Qufu

日期 DATE	日出 SUNRISE	日中	日落 SUNSET	昼长 DAYLENTH
2019年03月01日 周五	06:42:15	12:24:27	18:06:39	11:24:24
2019年03月02日 周六	06:40:57	12:24:15	18:07:34	11:26:37
2019年03月03日 周日	06:39:38	12:24:03	18:08:28	11:28:50
2019年03月04日 周一	06:38:19	12:23:50	18:09:22	11:31:03
2019年03月05日 周二	06:36:59	12:23:37	18:10:16	11:33:17
2019年03月06日 周三	06:35:38	12:23:24	18:11:09	11:35:31
2019年03月07日 周四	06:34:17	12:23:10	18:12:02	11:37:45
2019年03月08日 周五	06:32:55	12:22:55	18:12:55	11:40:00
2019年03月09日 周六	06:31:33	12:22:40	18:13:48	11:42:15
2019年03月10日 周日	06:30:10	12:22:25	18:14:40	11:44:30
2019年03月11日 周一	06:28:47	12:22:10	18:15:32	11:46:45
2019年03月12日 周二	06:27:24	12:21:54	18:16:24	11:49:00
2019年03月13日 周三	06:26:00	12:21:38	18:17:16	11:51:16
2019年03月14日 周四	06:24:36	12:21:22	18:18:07	11:53:31
2019年03月15日 周五	06:23:12	12:21:05	18:18:58	11:55:46
2019年03月16日 周六	06:21:47	12:20:48	18:19:49	11:58:02
2019年03月17日 周日	06:20:22	12:20:31	18:20:40	12:00:18
2019年03月18日 周一	06:18:57	12:20:14	18:21:31	12:02:34
2019年03月19日 周二	06:17:31	12:19:56	18:22:21	12:04:50
2019年03月20日 周三	06:16:06	12:19:39	18:23:11	12:07:05
2019年03月21日 周四	06:14:40	12:19:21	18:24:01	12:09:21

Original figures of western blot

Figure 1 western blot of ATP synthase in HG

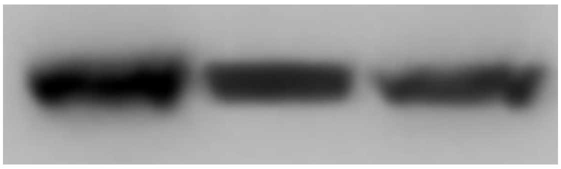

Figure 2 western blot of CS in HG

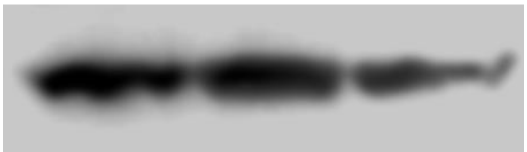

Figure 3 western blot of Drp1 in HG

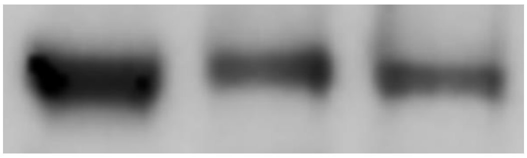

Figure 4 western blot of Mff in HG

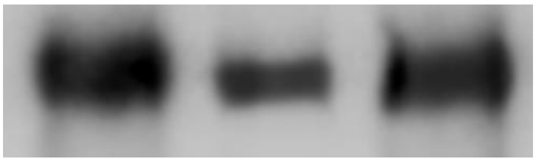

Figure 5 western blot of FIS1 in HG

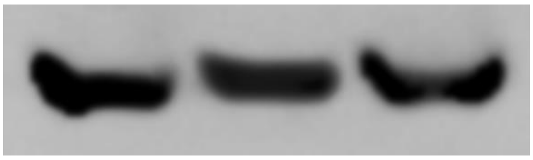

Figure 6 western blot of bax in HG

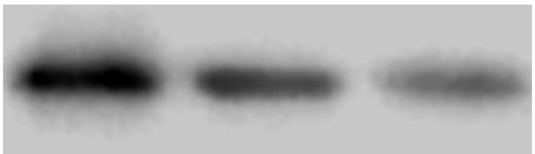

Figure 7 western blot of bcl2 in HG

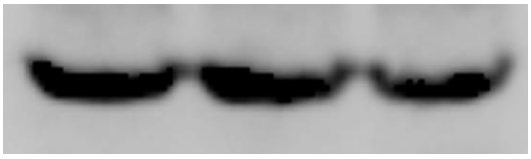

Figure 8 western blot of LCII in HG

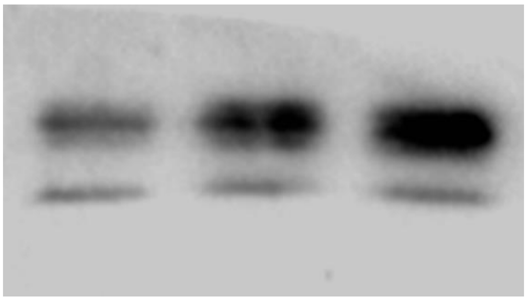

Figure 9 western blot of p62 in HG

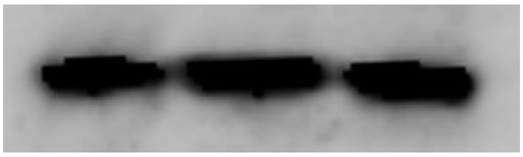

Figure 10 western blot of Cyto C in HG

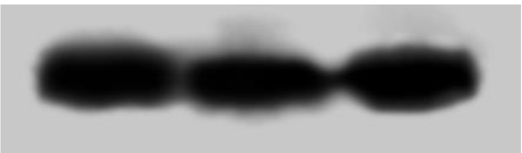

Figure 11 western blot of HIOMT in HG

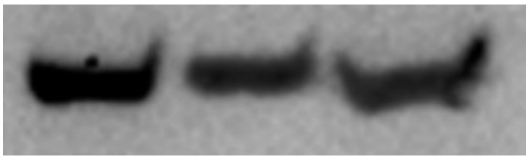

Figure 12 western blot of AANAT in HG

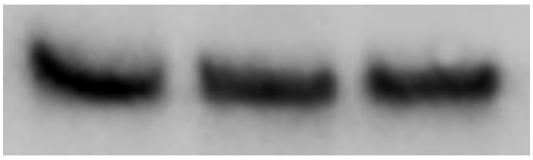

Figure 13 western blot of SS in HG

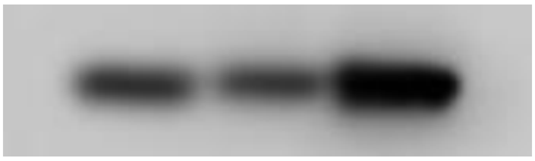

Figure 14 western blot of PC1 in HG

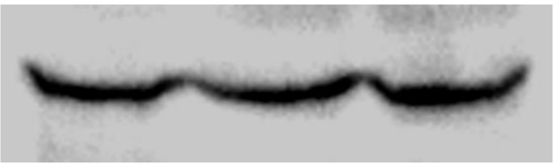

Figure 15 western blot of PC2 in HG

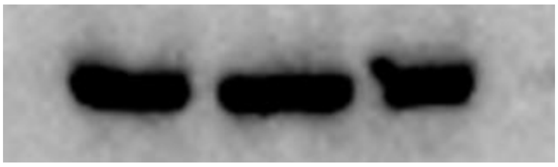

Figure 16 western blot of PAM in HG

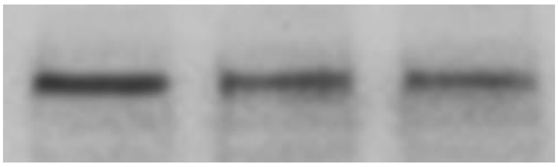

Figure 17 western blot of MLR in HG

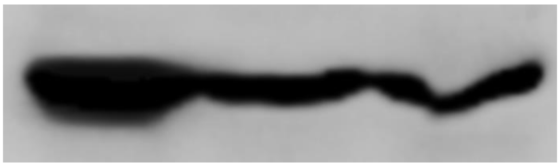

Figure 18 western blot of β-actin in HG

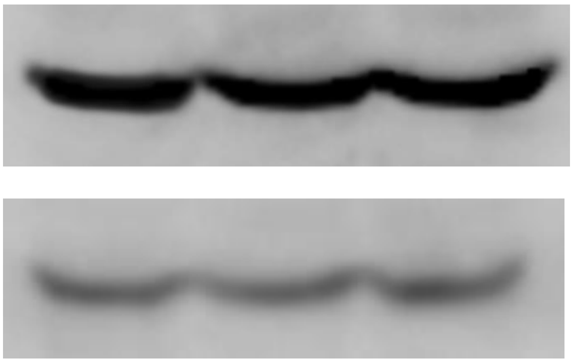

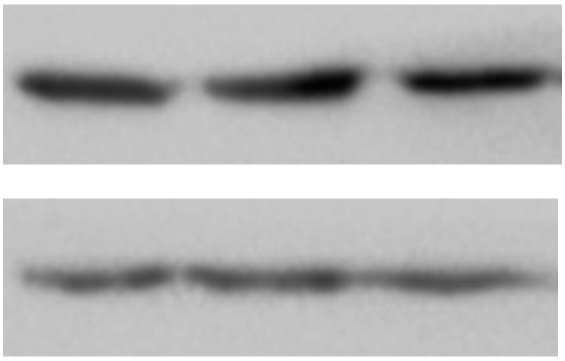

Supplement: Supplementary file 1 [file Data_Sheet_1.PDF]
